# Bovine milk-derived exosomes from colostrum are enriched with proteins implicated in immune response and growth

**DOI:** 10.1038/s41598-017-06288-8

**Published:** 2017-07-19

**Authors:** Monisha Samuel, David Chisanga, Michael Liem, Shivakumar Keerthikumar, Sushma Anand, Ching-Seng Ang, Christopher G. Adda, Ellen Versteegen, Markandeya Jois, Suresh Mathivanan

**Affiliations:** 1 Department of Physiology, Anatomy and Microbiology, School of Life Sciences, La Trobe University, Bundoora Victoria, 3086 Australia; 2 Department of Computer Science and Information Technology, School of Engineering and Mathematical Sciences, La Trobe University, Bundoora Victoria, 3086 Australia; 3 Department of Biochemistry and Genetics, La Trobe Institute for Molecular Science, La Trobe University, Melbourne Victoria, 3086 Australia; 4 Bio21 Institute, University of Melbourne, Victoria, 3010 Australia

## Abstract

Exosomes are extracellular vesicles secreted by multiple cell types into the extracellular space. They contain cell-state specific cargos which often reflects the (patho)physiological condition of the cells/organism. Milk contains high amounts of exosomes and it is unclear whether their cargo is altered based on the lactation stage of the organism. Here, we isolated exosomes from bovine milk that were obtained at various stages of lactation and examined the content by quantitative proteomics. Exosomes were isolated by OptiPrep density gradient centrifugation from milk obtained from cow after 24, 48 and 72 h post calving. As control, exosomes were also isolated from cows during mid-lactation period which has been referred to as mature milk (MM). Biochemical and biophysical characterization of exosomes revealed the high abundance of exosomes in colostrum and MM samples. Quantitative proteomics analysis highlighted the change in the proteomic cargo of exosomes based on the lactation state of the cow. Functional enrichment analysis revealed that exosomes from colostrum are significantly enriched with proteins that can potentially regulate the immune response and growth. This study highlights the importance of exosomes in colostrum and hence opens up new avenues to exploit these vesicles in the regulation of the immune response and growth.

## Introduction

Exosomes are membranous vesicles (30–150 nm) of endocytic origin that are secreted by multiple cell types under physiological and pathological conditions^1, 2^. These extracellular vesicles mediate intercellular communication by the transfer of various proteins, lipids and RNAs between different cell types^3^. Exosomes have been detected in all biofluids including blood, milk, saliva, urine, amniotic and bronchoalveolar lavage fluid^4^. Milk has been acclaimed as a highly composite nutrient system delineated during mammalian evolution to promote neonatal growth^5^. Although the proteome of bovine milk shows considerable difference from human milk, still both bovine milk as well as colostrum have been consistently studied due to their significant contribution in production of infant formula and protein supplements^6^. Colostrum is eminent as a nutrient packed fluid produced by mammary glands during the late stage of gestation immediately before parturition and is loaded with immune, development and tissue repairing factors^7^. An extensive range of proteins have already been identified in bovine colostrum which includes some highly abundant proteins like casein, β-lactoglobulin and α-lactalbumin, and low abundant proteins, such as monocyte differentiation antigen CD14 (CD14), glycosylation dependent cell adhesion molecule 1 (GLYCAM1), xanthine dehydrogenase/oxidase (XDH/XO), lactadherin (MFGE8) and clusterin (CLU)^8^. Apart from providing nourishment to the offspring, these proteins also play consequential role in modulating the immune system^9^.

Exosomes have recently been considered as major players in cell-cell communication^10^. It has been proposed that breast milk derived exosomes can be absorbed by the recipient tissue/cells and utilised in the fortification of the infant immune system^5^. There has been increasing evidences on the role of bovine milk exosomes as transporters of miRNAs for eliciting regulatory functions in the recipient cells^11^. The bovine milk exosome proteome has already provided novel information on milk protein composition and highlights the unrealized physiological importance of exosomes to mammary physiology^4^. Moreover, the cargo of exosomes have been shown to be altered by physical activity, feeding of cows and infection suggesting that milk exosomes could be exploited as reservoirs of biomarkers^4, 12^.

Previous proteomics studies have constantly focused on unfractionated colostrum and milk samples where low abundant and membrane proteins are often underrepresented^8, 13^. In this study, we isolated and characterised exosomes from healthy bovine colostrum obtained 24, 48 and 72 h after calving. In addition, exosomes were also isolated and characterised from cows in the mid-lactation stage referred to as mature milk. Quantitative proteomics and functional enrichment analysis of exosomes isolated from colostrum samples highlighted the enrichment of proteins implicated in immune response and growth. This study will not only help in unravelling the difference in the proteomic cargo of exosomes from colostrum to mid-lactation cows but also provide insights to the functional changes in mammary cells during various stages of lactation.

## Results

### Exosomal markers are enriched in fractions of density 1.08–1.22 g/mL

**Figure 1:**
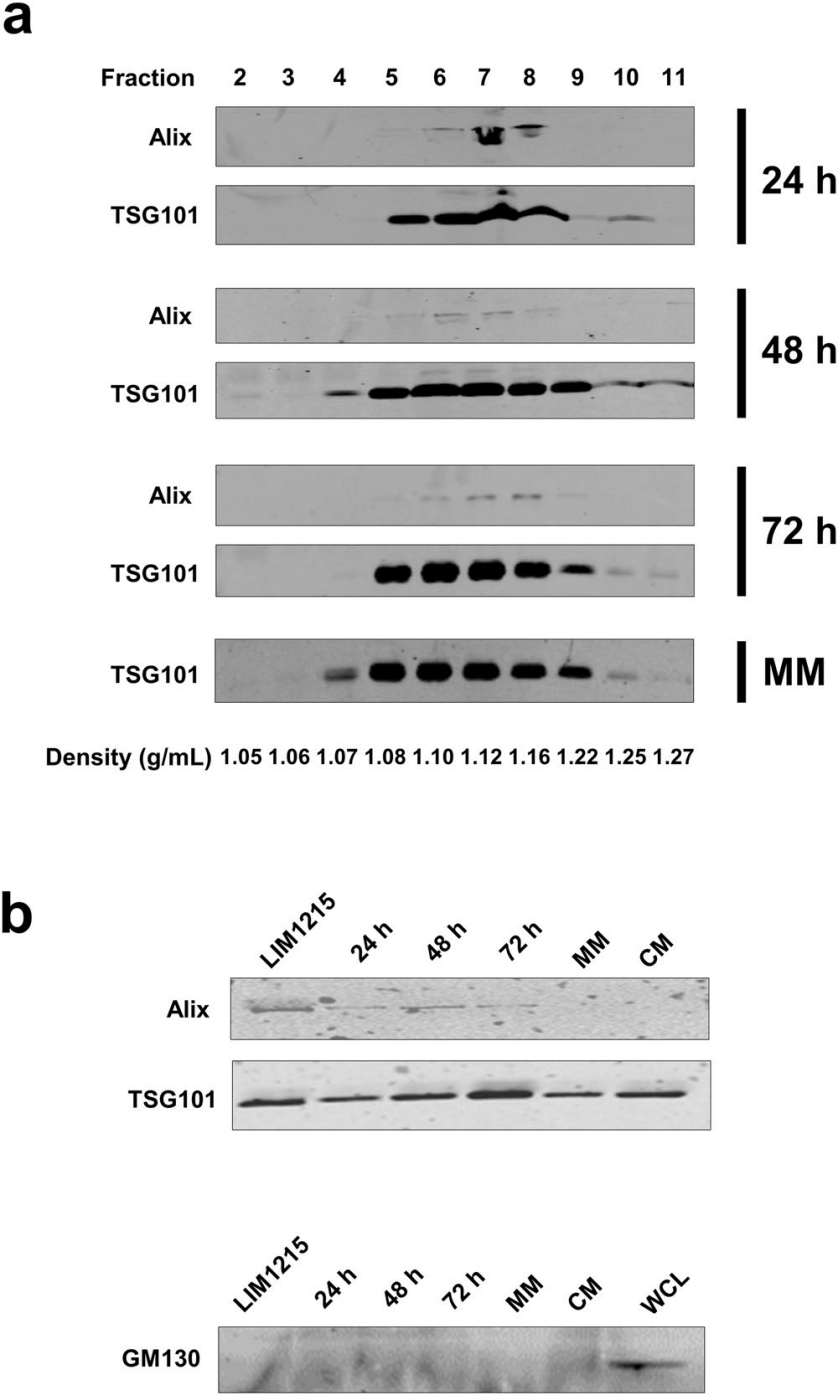
Western blot analysis of exosomes isolated from MM and colostrum. (**a**) Western blot analysis of fractions of increasing density obtained from the OptiPrep density gradient centrifugation which were probed for exosomal markers Alix and TSG101. TSG101 was identified in all the samples from fractions 5–9 corresponding to the buoyant density of 1.08–1.22 g/mL. The presence of Alix was limited to colostrum samples only. (**b**) Western blot analysis revalidating the presence of Alix in higher abundance only within colostrum exosomes as compared to mature milk (MM) and commercial milk (CM). TSG101 was found in all the samples including commercial milk exosomes. Exosomes isolated from LIM1215 colorectal cancer cells (LIM1215) was used as a positive control. GM130 was detected in LIM1215 colorectal cancer cell lysates (WCL) and was absent in the milk derived exosomal samples confirming the depletion of contaminating vesicles arising from apoptosis.

Exosomes were isolated by differential centrifugation coupled with ultracentrifugation from mid-lactation stage referred to as mature milk (MM) and colostrum samples after 24, 48 and 72 h of calving. To purify exosomes further, the crude exosomes obtained by ultracentrifugation from four different milk samples were separated using OptiPrep density gradient centrifugation^14^. To identify exosomes enriched samples, fractions obtained from density gradient centrifugation were subjected to Western blot analysis for exosomal enriched proteins Alix and TSG101^3^. Consistent for exosomes reported previously^4–7^, TSG101 was enriched in fractions 5–9 corresponding to the density of 1.08–1.22 g/mL in milk samples including MM, 24, 48 and 72 h (Fig. 1a). Alix was observed in fractions 6–8 in the three colostrum samples (Fig. 1a) but was not detected in any of the fractions in MM samples. In order to further confirm this observation, Western blot analysis was performed for Alix and TSG101 in exosomes isolated from colostrum samples, MM, commercial milk (CM) and LIM1215 colorectal cancer cells. Consistent with Fig. 1a, the amount of Alix was relatively low in MM and CM exosomes compared to colostrum samples (Fig. 1b). As fractions 6, 7 and 8 were enriched in exosomes, they were pooled together for further analysis.

### Biophysical analysis of exosomes isolated from colostrum and mature milk samples

**Figure 2:**
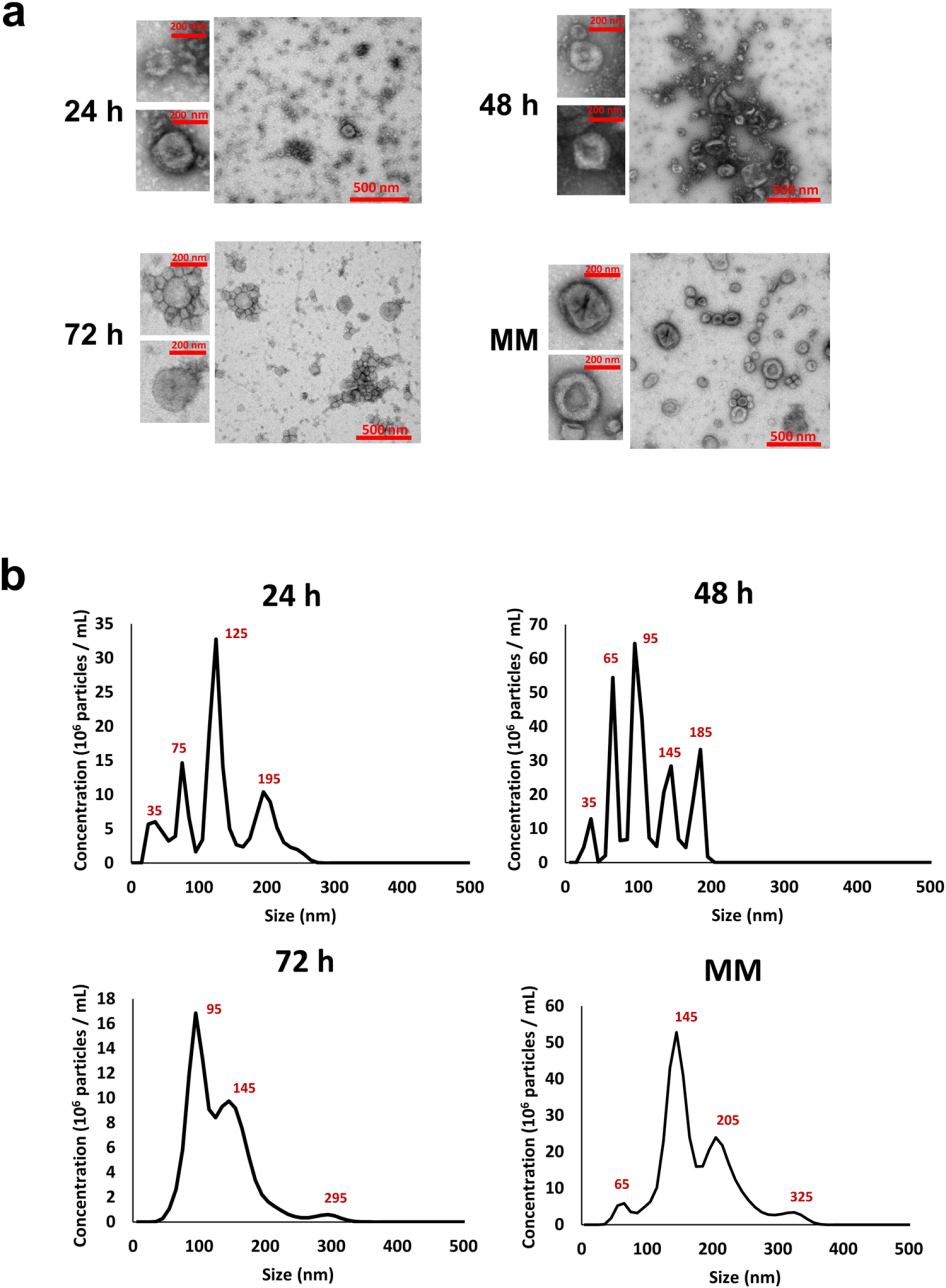
Biophysical characterization of exosomes isolated from MM and colostrum. (**a**) TEM images of exosomes isolated by OptiPrep density gradient centrifugation revealed vesicles with a morphology similar to exosomes and a diameter in the range of 30–150 nm for all four samples. The colostrum samples had meagrely scattered vesicles with higher protein background whereas the MM samples revealed a rich population of exosomes with typical cup-shaped morphology. (**b**) NTA revealed peak intensities to be 125, 95, 95 and 145 nm for 24, 48, 72 h colostrum and MM samples, respectively.

To characterize the isolated exosomes biophysically, transmission electron microscopy (TEM) and Nanoparticle Tracking Analysis (NTA) were performed. Vesicles that are characteristic of exosomes in the range of 30–150 nm in diameter were observed in all the four samples. The MM samples exhibited a rich profusion of mixed population of exosomes with predominantly intact vesicles consistent with classical exosome-like morphology (Fig. 2a). However, it was observed that the 24, 48 and 72 h colostrum samples contained sparsely scattered exosomes with higher levels of proteinaceous background presumably due to the high abundance of proteins in colostrum similar to plasma exosomes^15^ (Fig. 2a). All the four samples were also analysed using NTA (Fig. 2b). The peak intensities were detected to be 125, 95, 95 and 145 nm for 24, 48, 72 h colostrum and MM samples, respectively. The mean size of the vesicles derived from the MM sample were comparatively larger (173 nm) than those from colostrum samples (123 nm).

### Proteomic analysis of exosomes isolated from colostrum and mature milk

**Figure 3:**
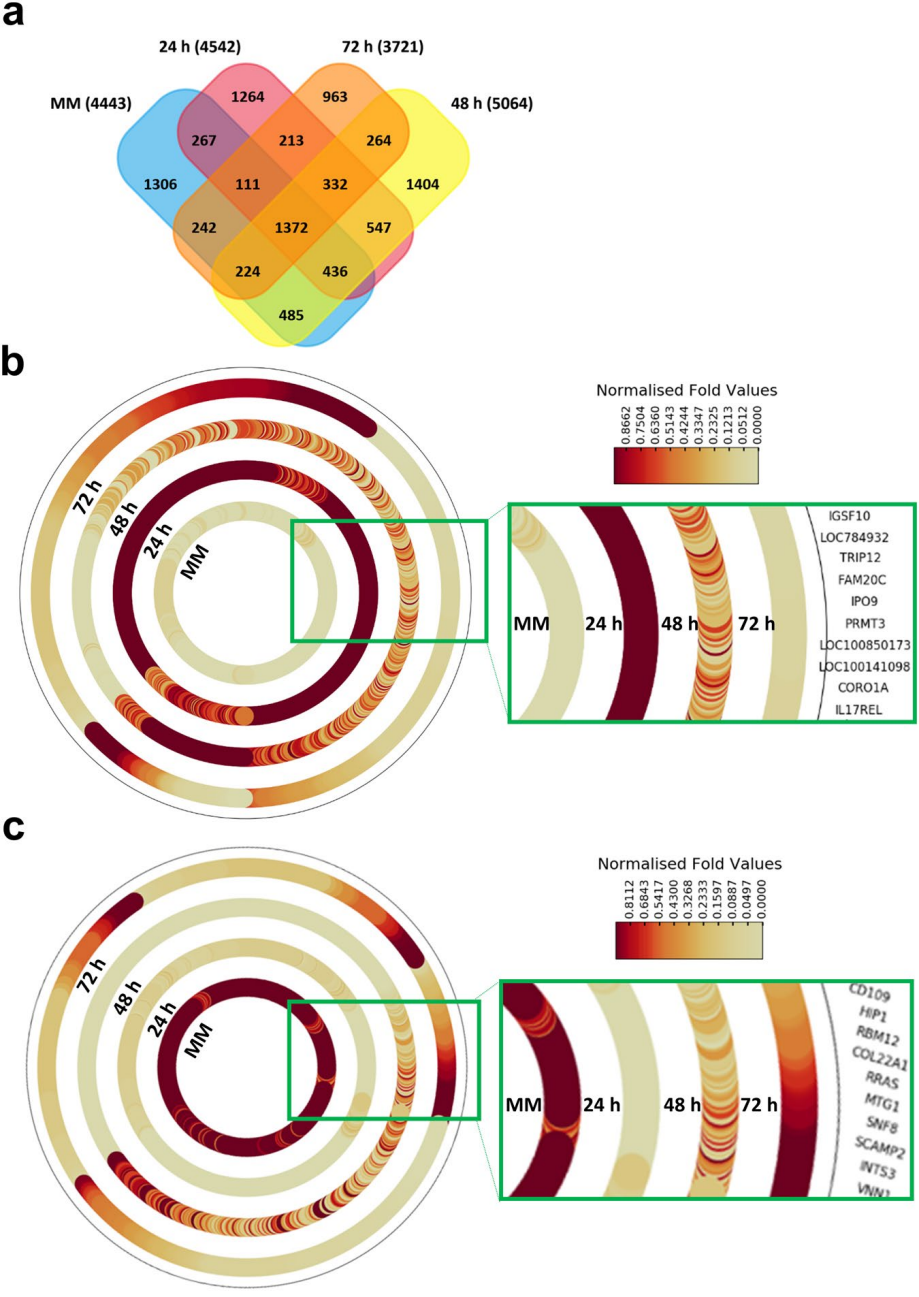
Proteomics analysis of exosomes isolated from MM and colostrum. (**a**) A four-way Venn diagram of proteins distributed between 24, 48, 72 h colostrum and MM exosome samples revealing 1372 proteins common to all dataset. (**b**) Heatmaps showing proteins that are 3-fold more abundant in exosomes isolated from 24 h colostrum samples compared to MM. A distinct pattern was revealed where proteins that were seen to be highly abundant in 24 h colostrum exosomes steadily diminished in 48 and 72 h colostrum exosomes and reached similar levels to the MM exosomes. (**b**) Proteins that are 3-fold more highly abundant in exosomes isolated from MM samples compared to 24 h colostrum sample were also plotted as a heatmap and revealed a similar distribution pattern.

Furthermore, the purified exosomes from colostrum and MM samples were analysed using LC-MS/MS-based label-free quantitative proteomics. Equal amounts of exosomal protein (30 µg) samples were separated by SDS-PAGE, gel bands were excised, reduced, alkylated and digested with trypsin. The extracted tryptic peptides were analysed on a LTQ-Orbitrap Elite mass spectrometer. The resulting MS/MS spectrum was analysed using X!Tandem against the *Bos taurus* RefSeq protein database and the protein list was consolidated (Supplementary Table S1). The data is deposited in ExoCarta^16^ and Vesiclepedia^17^. A total of 9430 unique proteins were identified in all four milk-derived exosomal samples. As shown in Fig. 3a, 1372 proteins were commonly identified in all four exosomes while 1264, 1404, 963 and 1306 proteins were unique to 24, 48, 72 h colostrum and MM samples, respectively. This huge variability in the proteomic content of the exosomes can be ascribed to difference in the lactation stage and/number of somatic cells in the milk^4^. To interpret the data quantitatively, label-free spectral counting-based quantitative proteomics analysis was performed on the MS data obtained from the colostrum and MM exosomes^14^ Proteins that are 3-fold highly abundant in exosomes isolated from 24 h colostrum samples compared with MM were plotted as a heatmap (Fig. 3b).

As shown in Fig. 3b, many proteins (Supplementary Table S2) are highly abundant in 24 h colostrum exosomes and are gradually depleted in 48 and 72 h colostrum exosomes saturating to levels similar to the MM exosomes (Fig. 3b). This observation is expected as it is generally thought that proteins that may be important in regulating the immune response and growth may be of high abundance in colostrum samples and are gradually saturated as the milk is produced regularly by the cow. Similarly, proteins that were 3-fold highly abundant in exosomes isolated from MM samples compared to the 24 h colostrum sample were plotted as a heatmap (Fig. 3c). Some of the abundant proteins in exosomes isolated from MM (Supplementary Table S3) were depleted in exosomes isolated from 24 h colostrum samples. A subset of these proteins in exosomes isolated from 48 and 72 h colostrum samples were of similar abundance to those from MM samples (Fig. 3c).

### Exosomal markers are enriched in colostrum samples

**Figure 4:**
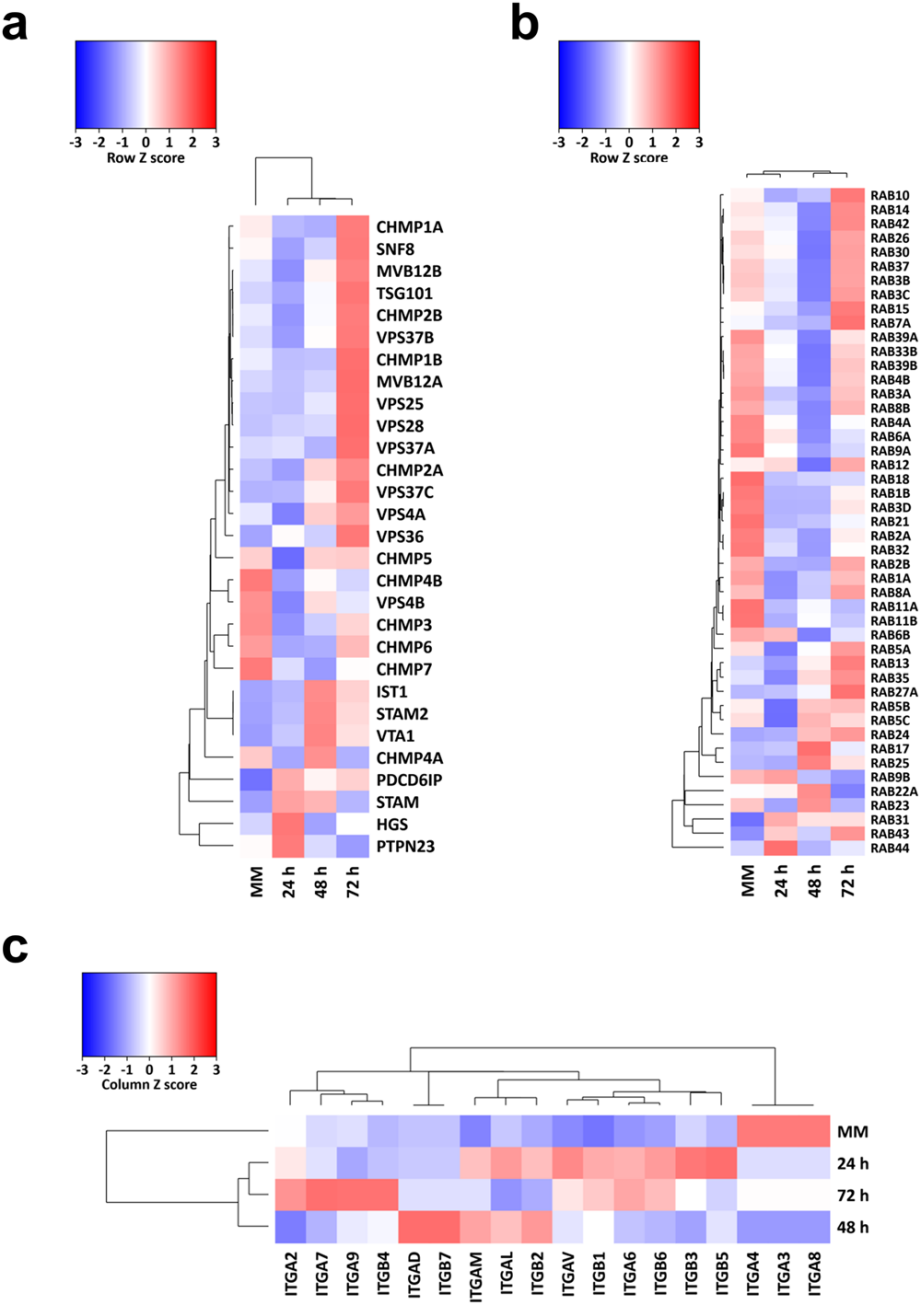
Exosomes from bovine milk are enriched with exosomal markers. (**a**) A heatmap showing enrichment of exosomal marker proteins in colostrum and MM exosomes. The scaled expression of each protein, denoted as the row *Z-score*, is plotted in the red–blue colour scale. High expression levels are indicated in red and low expression levels are shown in blue. Similar to western blot analysis, Alix (PDCD6IP) was found to be of higher abundance in the colostrum samples when compared with the MM samples. The 72 h colostrum samples had a higher level of many of the ESCRT component proteins. (**b**) Higher abundance of Rab family proteins was observed in both colostrum and MM exosomes. (**c**) An enrichment of integrins, which play a crucial role in exosomal uptake by target cells was observed in all the exosomes.

In order to evaluate the abundance of known exosomal enriched proteins in the colostrum and MM exosomes, the quantitative proteomic data was plotted as a heatmap for ESCRT components, Rab GTPases and integrins. As shown in Fig. 4a, consistent with the Western blot analysis (Fig. 1b), the ESCRT accessory protein Alix (PDCD6IP) was found to be of higher abundance in the colostrum samples compared to MM samples. Many of the ESCRT component proteins including CHMP and VPS family proteins were enriched in 72 h colostrum samples. Furthermore, all the four exosomes were highly enriched with Rab GTPases that are implicated in exosome biogenesis, vesicular trafficking and fusion^18^ (Fig. 4b). Similarly, integrins that are critical for the uptake of exosomes by target cells were enriched in exosomes (Fig. 4c) from the colostrum and MM^19^. However, the exact subtype of integrin molecule differed between the exosomes. Some common proteins associated with milk, like Butyrophilin, Xanthine oxidase, Adipophilin and Lactadherin (MFG-E8) were also present in higher abundance in the exosomes.

### Exosomes from colostrum are enriched with proteins implicated in immune response

**Figure 5:**
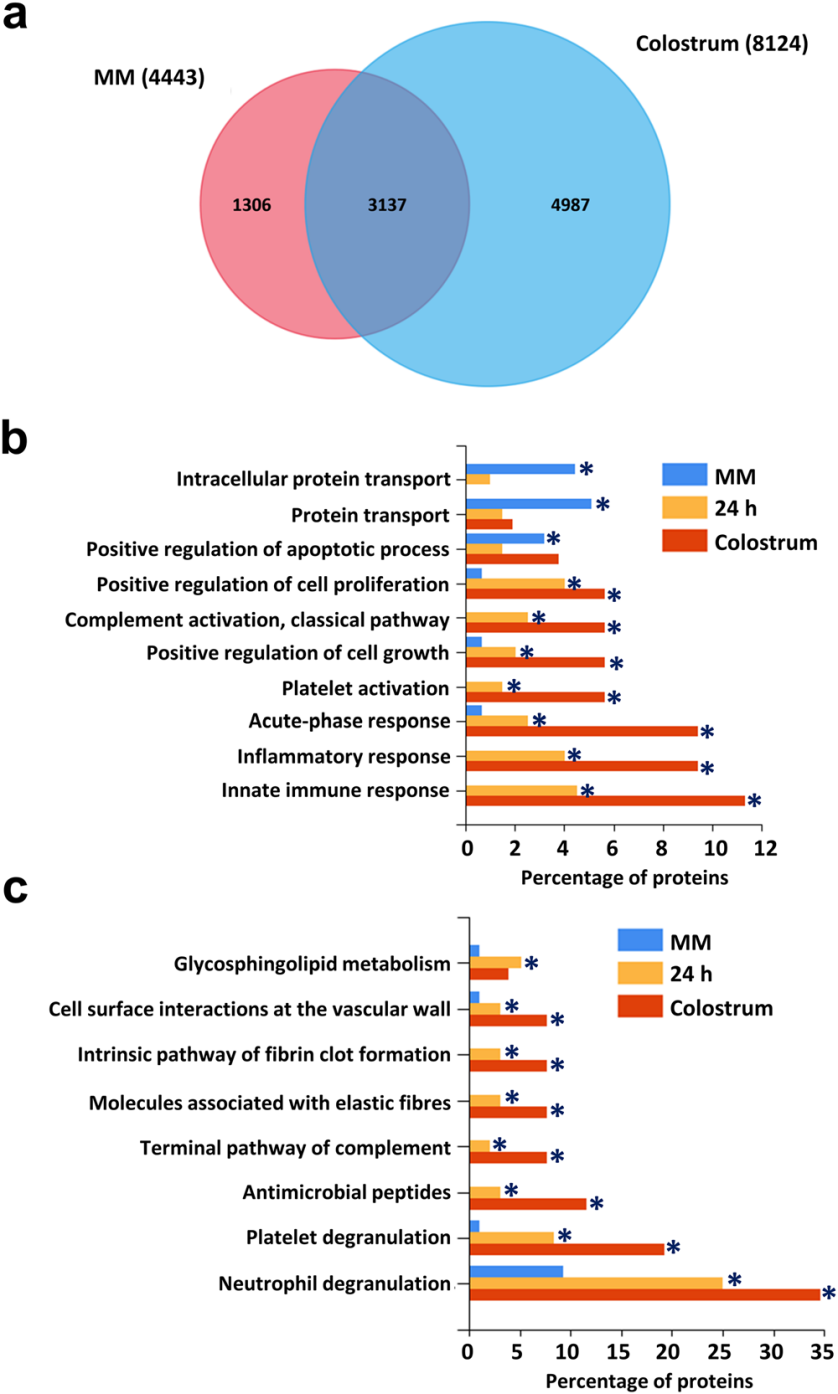
Exosomes from colostrum are enriched with proteins implicated in immune response. (**a**) Venn diagram of proteins identified in MM and colostrum exosomes. More than 4980 proteins were uniquely identified in colostrum samples. (**b**) Functional enrichment analysis using FunRich revealed various proteins associated with innate immune response, inflammatory response, acute-phase response, platelet activation, and cell growth were highly enriched in exosomes isolated from colostrum (24, 48 and 72 h sample) along with significant enrichment in 24 h sample on its own. * Denotes P < 0.05. (**c**) Histogram representing proteins related to complement activation and antimicrobial peptides were significantly enriched in exosomes from colostrum and 24 h sample when compared with MM. * Denotes P < 0.05.

To gain more insights from the proteomic data, proteins identified from exosomes isolated from 24, 48 and 72 h colostrum samples were combined into one master list (referred to as colostrum from now on). The combined colostrum exosome protein data was compared with the MM exosome proteome (Fig. 5a). A total of 8124 and 4443 unique proteins were identified in each of the combined colostrum and MM exosomal proteomes. A vast majority of the proteins (3137) were identified in common between the MM and combined colostrum exosomal proteomes. Proteins that were at least 3-fold highly abundant in the combined colostrum and MM exosomal proteome were further subjected to functional enrichment analysis using the FunRich tool^20^. Proteins that were at least 3-fold highly abundant in the 24 h colostrum samples compared to MM were also used in this analysis. As shown in Fig. 5b, exosomes from colostrum samples were significantly enriched with proteins implicated in the innate immune response, inflammatory response, acute-phase response, platelet activation, cell growth and complement activation. In contrast, exosomes from MM were enriched with proteins implicated in transport and apoptosis. The analysis of the biological pathways in exosomes isolated from colostrum samples highlighted the enrichment of proteins implicated in platelet/neutrophil degranulation, antimicrobial peptides, fibrin clot formation, elastic fibres and complement activation. Overall, these data suggest that exosomes in colostrum are enriched with proteins implicated in immune response and growth. Hence, it can be speculated that exosomes in colostrum may have a significant role in regulating the immune response and growth in infants, at least in part.

## Discussion

Over the past, there has been growing interest to elucidate the multifaceted framework of the milk proteome. Hence, various techniques have been used to fractionate and identify these proteins in both colostrum and MM samples. The complex nature of milk has not allowed greater depth of proteome coverage as most of the studies have focused on unfractionated milk samples. Despite a significant number of studies pertaining to understanding the properties of bovine colostrum, there has been very few studies that strive to highlight the change from colostrum to mature milk, at various time points. Recently, Zang *et al*. for the first time showed the quantitative changes that occur in the bovine milk proteome in the first 9 days of calving^8^. Understanding this transition is highly crucial in order to decipher the complexity in the interactions of milk proteins that have potential impact on the development of the infant.

The aim of the present study was to investigate, characterize and compare the bovine milk exosome proteome from MM and colostrum samples after 24, 48 and 72 h of calving. We used OptiPrep density gradient centrifugation to purify the exosomes. All the populations of exosomes isolated expressed the exosomal marker TSG101, whereas Alix was found to be in higher abundance in colostrum samples. The exosomes revealed typical cup shaped morphology and equilibrated within the same proposed density of 1.08–1.24 gm/mL^15^. Comparative proteomic analysis revealed common as well as many unique proteins among the samples. As expected exosomes from all the four samples were highly enriched with some common milk proteins like Butyrophilin, Xanthine oxidase, Adipophilin and Lactadherin (MFG-E8). Various proteins playing a major role in vesicle docking and fusion, including the well-known Rab family proteins, were also present in abundance^18^. Moreover, integrins responsible for anchorage and uptake of exosomes were observed in amplitude within all the four samples. A distinct pattern emerged within the samples when 3-fold more abundant proteins in the 24 h sample were compared to MM. It was observed that certain proteins that were highly abundant in 24 h, slowly diminished at 48 and 72 h and were at similar amounts to the MM sample. Previously, certain immune-related proteins in bovine milk have been reported to decrease with time^6, 21, 22^. This phenomenon is evident in the exosome proteome as well indicating that exosomes could be utilised as sources of potential biomarkers for quality control of milk.

Recent evidences has shown that exosomes play an integral role in activating immune function and human breast milk exosomes have already been seen to impact the immune system of the infant^23^. The enrichment analysis using FunRich^24^ showed that exosomes from bovine colostrum samples were significantly enriched with proteins associated with the innate immune response. Proteins involved in the foremost systemic reaction-like acute phase response (APR) were highly upregulated in the colostrum samples. The acute phase proteins modulate the blood plasma composition and are known to be favorable to the organism by averting microbial growth and reestablishing homeostasis. Some microorganisms are prone to opsonisation by acute phase proteins which can also illicit complement activation^25^. Various antimicrobial peptides were also found in higher abundance along with inflammatory response and complement activation proteins. Their presence is critical, as they are first line of defense during the growth of the infant. These complement proteins are in balance with other immunological and non-immunological protective mechanisms on the gastrointestinal lining in the infant. Proteins regulating cell growth and positive regulation of cell proliferation were also enriched in the colostrum samples. These proteins regulate the progress of the newborn’s immune system along with differentiation, repair and growth of various infant-specific organs and target tissues while preserving the functionality of the lactating mammary gland^26^. The abundance of proteins regulating blood coagulation along with platelet activation indicate the distinguished role of colostrum in modulating the innate immune system of the infant.

Although the bovine milk exosome proteome has been previously studied^4^ it was focused on milk obtained from a mid-lactating cow. This study provides a novel in-depth functional analysis of colostrum in which we have used a proteomics approach to gather biologically germane information about the functional role of bovine colostrum exosomal milk proteins. It is possible that some of the proteins identified in this study could be adsorbed to the outer membrane surface of the exosomes. Nevertheless, the results suggest that a series of proteins present in the colostrum exosome proteome have a fundamental role in cellular growth and repair as well as innate immune responses against pathogen infections. Furthermore, the results indicate that various proteins present in colostrum modulate the proliferative events within the gastrointestinal tract of the infant, including blood cell development. This data highlights the biological role of bovine milk exosomes which contain a rich plethora of proteins that are not only involved in immune regulation, but also in the growth, repair and development of the infant.

## Materials and Methods

### Milk samples

Autumn calving dairy cows were selected at random in this study. Dairy cows were Holstein Friesian bred and resided at the Warnock Family Dairy Farm in Swanpool in North Eastern Victoria, Australia. The cows used in the study had moderate activity level and were free of any disease. La Trobe Animal Ethics committee approved all experimental protocol used in this study. All methods were performed in accordance with the relevant guidelines and regulations. Prior to the study, all cows were healthy. Bovine colostrum samples were individually collected within 24 (first two milking of 12 and 24 h pooled together), 48 and 72 h of calving. Mature milk (MM) samples were also collected for comparative analysis. Autumn calving dairy cows were selected at random for this study. Milk samples were frozen immediately after milking and were stored at −80 °C until use.

### Isolation of exosomes by differential centrifugation and ultracentrifugation

The individual milk samples obtained from five cows at three different time points (24, 48 and 72 h of calving) were pooled. Milk samples were centrifuged first at 5,000 *g* for 30 min at 4 °C and the floating milk fat pellet was removed. For the removal of casein and other cell debris, the skimmed milk samples were then subjected to three successive centrifugations at 4 °C for 1 h each at 12,000 *g*, 35,000 *g* and 70,000 *g*. This was followed by a final 100,000 *g* spin using a SW28 rotor (Beckman Coulter instruments, Vic, Australia). The precipitates obtained were resuspended in 0.5 mL PBS and stored at −80 °C.

### Density gradient ultracentrifugation

Density gradient ultracentrifugation was performed as described previously^14^. A discontinuous iodixanol gradient consisting of 40% w/v, 20% w/v, 10% w/v, and 5% w/v solutions of iodixanol was prepared by diluting a stock solution of OptiPrep (60% w/v aqueous iodixanol from Sigma life Sciences) in 0.25 M sucrose/10 mM Tris, pH 7.5. The gradient was set up in a 12 mL polyallomer tube (Beckman Coulter) by subsequent layering of 3 mL fractions of 40%, 20%, 10% iodixanol solution and 2.8 mL of 5% iodixanol solution. The exosomal pellet obtained by ultracentrifugation from milk samples was layered on top of a 5% iodixanol solution and centrifuged at 100,000 *g* for 18 h at 4 °C. Starting from the top of the tube, 12 individual 1 mL gradient fractions of increasing density were collected and fractions were diluted with 1.5 mL PBS followed by centrifugation at 100,000 *g* for 1 h at 4 °C. The resulting pellet obtained was further washed with 1.5 mL PBS, centrifuged at 100,000 *g* for 1 h at 4 °C and resuspended in 200 µL PBS. A control OptiPrep gradient column was overlaid with 500 µL of 0.25 M sucrose/10 mM Tris, pH 7.5 was used in parallel to determine the density of each fraction.

### Electron microscopy

Exosome samples were diluted to a protein concentration 0.2 mg/ml in PBS and 10 μL applied to 400 mesh copper grids that were coated with a thin layer of carbon for 2 min. Excess material was removed by blotting and samples were negatively stained twice with 10 μL of a 2% uranyl acetate solution (w/v; Electron Microscopy Services). The grids were air dried and viewed using a JEOL JEM-2100 transmission electron microscope operated at 200 kV.

### SDS-PAGE and tryptic digestion

Equal amounts of milk proteins obtained from exosome samples (30 µg) were loaded onto precast NuPAGE^®^ 4–12% Bis-Tris gels in 1x MES SDS running buffer. Gels were run at a constant voltage of 150 V followed by visualization of proteins with Coomassie stain (Bio-Rad). The staining was performed for 1 h and destained in 20% ethanoic acid and 7.5% acetic acid in Milli-Q water overnight. Gel bands (20) were excised and subjected to in-gel reduction, alkylation and trypsinization, as previously described^27^. Briefly, gel bands were reduced with 10 mM DTT (Bio-Rad) for 30 min, alkylated for 20 min with 25 mM iodoacetamide (Sigma), and subsequently digested overnight at 37 °C with 15 ng of sequencing grade trypsin (Promega). Digested peptides were further extracted with 100 μL 50% (v/v) acetonitrile, 0.1% trifluroacetic acid, freeze dried and resuspended in 30 µL 3% (v/v), 0.1% trifluroacetic acid and analysed by liquid chromatography mass spectrometry (LC-MS/MS).

### LC-MS/MS

LC MS/MS was carried out on a LTQ Orbitrap Elite (Thermo Scientific) with a nanoESI interface in conjunction with an Ultimate 3000 RSLC nanoHPLC (Dionex Ultimate 3000). The LC system was equipped with an Acclaim Pepmap nano-trap column (Dionex-C18, 100 Å, 75 µm x 2 cm) and an Acclaim Pepmap RSLC analytical column (Dionex-C18, 100 Å, 75 µm × 15 cm). The tryptic peptides were injected to the enrichment column at an isocratic flow of 5 µL/min of 3% v/v CH_3_CN containing 0.1% v/v formic acid for 5 min before the enrichment column was switched in-line with the analytical column. The eluents were 0.1% v/v formic acid (solvent A) and 100% v/v CH_3_CN in 0.l% v/v formic acid (solvent B). The flow gradient was (i) 0–5 min at 3% B, (ii) 5–25 min, 3–25% B (iii) 25–27 min, 25–40% B (iv) 27–29 min, 40–80% B (v) 29–31 min at 80% B (vii) 31–32 min, 80–3% B and (viii) 32–38 min at 3% B. The LTQ Orbitrap Elite spectrometer was operated in the data-dependent mode with nanoESI spray voltage of 1.8 kV, capillary temperature of 250 °C and S-lens RF value of 55%. All spectra were acquired in positive mode with full scan MS spectra scanning from m/z 300–1650 in the FT mode at 240,000 resolution after accumulating to a target value of 1.0e^6^. Lock mass of 445.120025 was used. The top 20 most intense precursors were subjected to rapid collision induced dissociation (rCID) with normalized collision energy of 30 and activation q of 0.25. Dynamic exclusion with of 30 seconds was applied for repeated precursors.

### Database searching and protein identification

Peak lists were generated using extract-msn as part of Bioworks 3.3.1 (Thermo Scientific) using the following parameters: minimum mass 300; maximum mass 5,000; grouping tolerance 0.01 Da; intermediate scans 200; minimum group count 1; 10 peaks minimum and total ion current of 100. Peak lists for each LC-MS/MS run were merged into a single mascot generic format. Automatic charge state recognition was used because of the high resolution survey scan (30,000). LC-MS/MS spectra were searched against the NCBI RefSeq bovine protein database^28^ using X!Tandem Sledgehammer (2013.09.01.1). Search parameters used were: fixed modification (carboamidomethylation of cysteine; +57 Da), variable modifications (oxidation of methionine; +16 Da and N-terminal acetylation; +42 Da), three missed tryptic cleavages, 20 ppm peptide mass tolerance and 0.6 Da fragment ion mass tolerance.

### Label-free spectral counting

The relative protein abundance between the samples was obtained by estimating the ratio of normalized spectral counts (RSc) as previously described^29^.

$${\rm{RSc}}\,{\rm{for}}\,{\rm{protein}}\,{\rm{A}}=[(sY+c)(TX-sX+c)/(sX+c)(TY-sY+c)]$$

Where *s* is the significant MS/MS spectra for protein A, *T* is the total number of significant MS/MS spectra in the sample, *c* is the correction factor set to 1.25, and *X* or *Y* are the exosome samples at different time points (24 h, 48 h, 72 h and MM). When RSc is less than 1, the negative inverse RSc value was used.

### Functional enrichment analysis

Functional enrichment analysis was performed using FunRich tool. The heatmap for the proteomic data was obtained from FunRich. Statistical analysis for gene set enrichment was performed using inbuilt analysis tools in FunRich.

All the experimental methods were performed in accordance with the institutional National and International guidelines and regulations.

## Electronic supplementary material

Dataset 1

Dataset 2

Dataset 3
